# Event-Based Gesture Recognition through a Hierarchy of Time-Surfaces for FPGA

**DOI:** 10.3390/s20123404

**Published:** 2020-06-16

**Authors:** Ricardo Tapiador-Morales, Jean-Matthieu Maro, Angel Jimenez-Fernandez, Gabriel Jimenez-Moreno, Ryad Benosman, Alejandro Linares-Barranco

**Affiliations:** 1 Robotics and Technology of Computers Lab (ETSII-EPS), University of Seville, 41089 Sevilla, Spain; ajimenez@atc.us.es (A.J.-F.); gaji@atc.us.es (G.J.-M.); alinares@atc.us.es (A.L.-B.); 2 aiCTX AG, 8092 Zurich, Switzerland; 3 Neuromorphic Vision and Natural Computation, Sorbonne Université, 75006 Paris, France; corr@jmatthi.eu (J.-M.M.); ryad.benosman@upmc.fr (R.B.); 4 SCORE Lab, Research Institute of Computer Engineering (I3US), University of Seville, 41089 Seville, Spain

**Keywords:** dynamic vision sensors, event-based, synchronous digital VLSI, HDL, FPGA, pattern recognition, AER

## Abstract

Neuromorphic vision sensors detect changes in luminosity taking inspiration from mammalian retina and providing a stream of events with high temporal resolution, also known as Dynamic Vision Sensors (DVS). This continuous stream of events can be used to extract spatio-temporal patterns from a scene. A time-surface represents a spatio-temporal context for a given spatial radius around an incoming event from a sensor at a specific time history. Time-surfaces can be organized in a hierarchical way to extract features from input events using the Hierarchy Of Time-Surfaces algorithm, hereinafter HOTS. HOTS can be organized in consecutive layers to extract combination of features in a similar way as some deep-learning algorithms do. This work introduces a novel FPGA architecture for accelerating HOTS network. This architecture is mainly based on block-RAM memory and the non-restoring square root algorithm, requiring basic components and enabling it for low-power low-latency embedded applications. The presented architecture has been tested on a Zynq 7100 platform at 100 MHz. The results show that the latencies are in the range of 1 μs to 6.7 μs, requiring a maximum dynamic power consumption of 77 mW. This system was tested with a gesture recognition dataset, obtaining an accuracy loss for 16-bit precision of only 1.2% with respect to the original software HOTS.

## 1. Introduction

Pattern recognition is one of the most important challenges in artificial vision. Inside the field of frame-based vision, Convolutional Neural Networks (CNNs) have become one of the most powerful approaches to extract features from images [1], due to their relatively simple supervised training and high accuracy they obtain. However, this kind of networks performs typically millions of multiplication and accumulation operations (MAC) [2,3], and also redundant operations (e.g., multiply by 0). Therefore, they are usually trained and deployed in graphic processor units (GPUs) or high performance computing (HPC) servers [4], which usually have a high power consumption. Regarding the sensors, frame-based cameras have to scan all the pixels of the captured scene within a fixed time period. Most of these events have not changed with respect to the previous frame, which has an important impact on computation, latency and memory consumption, since all the pixels must be stored and processed. In addition to CNNs, there are other pattern recognition techniques, such as K-nearest neighbours (K-NN) [5,6], which are easier to implement and do not require any kind of training. However, their accuracy depends on the particular population to be compared each time, since these techniques are not flexible and dynamically adaptable to each new case. Therefore, their complexity increases with the number of output classes of the classifier, and thus more computation and power are needed.

In recent years, other technologies, such as neuromorphic engineering, have reduced the number of operations and their power consumption, by optimising the process of information transmission, which takes inspiration from the biological nervous system [7]. Neuromorphic engineering mimics the way in which the human brain processes information, which is a hierarchy of layers of neurons connected typically in a feed-forward way with some recurrent connections [8,9]. In this paradigm, information is encoded in spikes (events), which are processed by these neurons. There are event-based sensors for audio [10,11] and visual information processing, such as the Dynamic Vision Sensor [12], and others that came after this, such as Time-to-First Image Sensor [13], Asynchronous Time-based Image Sensor [14], Sensitivity DVS [15], Dynamic Active Vision Sensor [16], Change-driven vision sensor [17] or Samsung DVS [18], among others. These neuromorphic vision sensors operate in a different way from frame-based cameras. Each pixel of these sensors is independent and generates an event when the luminosity of a scene has changed in time over a threshold. Therefore, if there are no changes in a pixel, events are not transmitted. Due to the independence among pixels, these sensors provide a continuous representation of the information.

Several works have processed the stream output of these sensors using spiking neural networks (SNN) to solve several tasks, such as object tracking [19,20,21], approaching detection [22], or shape recognition [23,24].

Recent works, as presented in [25], adapted the concept of CNNs to the event-based domain, being trained with specialised datasets to classify different objects, such as poker pips [26] or N-MNIST [27]. These solutions implement the Leaky-Integrate-and-Fire neuron model (LIF) to extract features for the incoming streams [28], which requires a considerable amount of resources to emulate the neuron model for a given relatively large CNN.

Other works, such as the Hierarchy Of Time Surfaces (HOTS) [28], consider only the activity of recent past events, creating a low level feature representation. The HOTS algorithm makes use of rich information provided by the stream of events, creating time-surfaces, which represent the recent temporal activity for a spatial neighbourhood.

Currently, there are several neuromorphic platforms, such as Spinnaker [29], BrainScale [30], IBM TrueNorth [31], AICTX Dynap-SE [32] and Intel Loihi [33] to deploy this kind of techniques. These hardware platforms can implement scalable networks connecting multiple boards with a maximum of 460 million neurons and 460 billion synapses with power consumptions between 100 mW and 50 kW [34]. However, such solutions are not ideal for embedded systems, due to their size, latency and power consumption. In contrast, FPGAs have become one of the best platforms to implement real-time solutions for event-based algorithms, due to their re-configurability, parallelism and relatively lower power consumption compared to other platforms, even for embedded systems.

In this context, our motivation is to explore the FPGA implementation of the HOTS algorithm in the search of a low-power solution for real-time embedded system applications. In this work, we present a novel FPGA architecture to accelerate a HOTS algorithm implementation. The main aims of this architecture are to reduce the power consumption and to speed up the computation, for its applicability to embedded solutions in fields such as robotics. Exploiting architectural benefits from FPGAs, such as block-RAM and parallelism, our solution integrates some other artifacts, such as non-restoring square-root procedure, for reducing latency and power consumption. This architecture has been tested through a gesture recognition demo implementation in real time.

The contributions of this paper can be summarized as:

- HDL description and implementation of HOTS for FPGA, taking advantage of their memory organization and square-root algorithms.
- Real-time demonstration for embedded systems and proof of their low latency and reduced power consumption.

The paper is organized as follows: Section 2.1 describes the event-based sensors paradigm, Section 2.2 explains the time-surface concept, Section 2.3 presents the hardware architecture and describes the main modules in detail. Finally, the experimental results and conclusions are presented in Section 3 and Section 4, respectively.

## 2. Materials and Methods

### 2.1. Event-Based Vision Sensors

**Figure 1:**
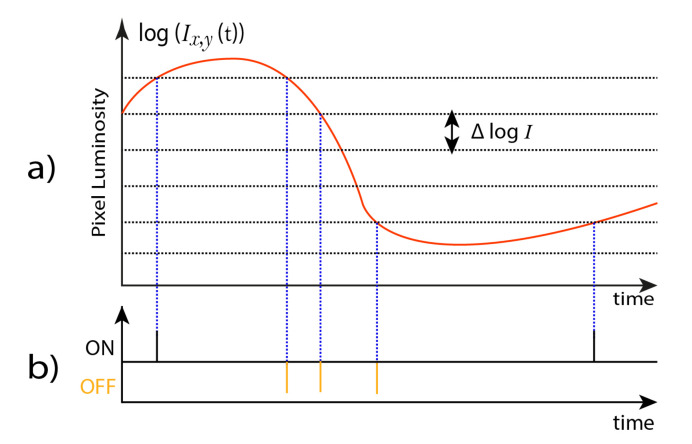
ATIS operation principles. When a pixel’s luminosity change reaches a given threshold (**a**), it produces a visual event with an (x,y) address and a polarity, which is either ON or OFF (**b**).

In this work, visual information obtained from the Asynchronous Time-based Image Sensor (ATIS) [14] and the Dynamic Vision Sensor (DVS) [12] were used. These sensors capture a dynamic reality, where each pixel triggers an event when the change in the luminosity exceeds a threshold. This luminosity change is encoded in the polarity (*p*) of the visual event, which can be ON (*p* = 1) when the luminosity increases, and OFF (*p* = 0) when the luminosity decreases. Therefore, static visual scenes will not produce any events, since there are no changes in them. In other words, if there are not moving activity, these sensors do not generate any events, avoiding the processing of redundant data. The behaviour of the ATIS sensor is shown in Figure 1.

ATIS and DVS sensors have a spatial range of 304 × 240 and 128 × 128, respectively, and a high temporal resolution in the order of milliseconds, which allows capturing highly fast dynamical scenes. The output of this kind of sensors is a stream of sparse events.

**Figure 2:**
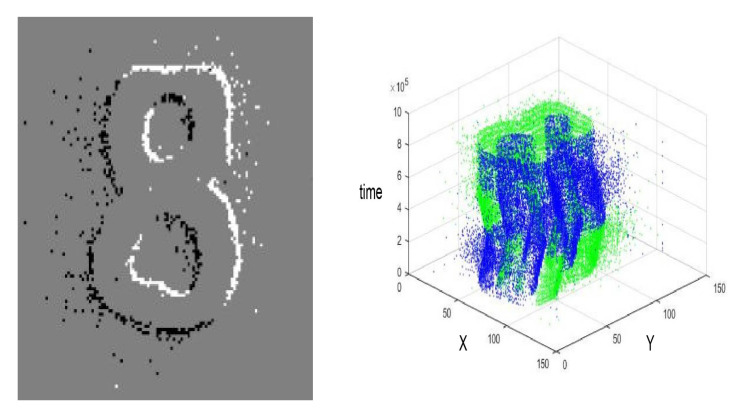
**Left**: captured histogram of events from neuromorphic vision sensors (ON = black events; OFF = white events). **Right**: Events temporal activity diagram from the sensor.

An event can be defined as in Equation (1):

(1) e=[x,y,p,t]

where (x,y) represents the spatial position of the event, *p* its polarity and *t* the timestamp. This representation is also referred to as Address-Event-Representation (AER), together with an asynchronous REQ and ACK protocol. The output events of these sensors are commonly used by a software system, such as jAER [35], in order to implement any kind of software filters or algorithms. In this work, these events were used to create time-surfaces on FPGA that were later used to extract patterns from the input stimulus. Figure 2 shows an example of events captured by these sensors and their activity along time.

### 2.2. Time-Surfaces

The time-surface concept was introduced in [28]. A time-surface describes the spatial-temporal activity of a neighbourhood around a received event $e_{k}$. Its calculation is based on the time context concept. The time context $T_{k}(u,p)$ of the event $e_{k}$ is defined as a square matrix of timestamps obtained from a neighbourhood of the difference between the timestamp $t_{k}$ of the incoming event $e_{k}$ and the timestamps of the most recent neighbours, for the polarity *p*. This matrix has a dimension of (2R+1)×(2R+1) and it is centered on $e_{k}$, of spatial coordinate (xk,yk). This can be mathematically expressed as in Equation (2):

(2) $$T_{k}\left(u,p\right)=\left\{t_{k}-t\right\}:t=max\left\{t_{j}\right\}\forall e_{j}=\left[x_{j},y_{j},p,t_{j}\right]\in \left\{e_{k}+u\right\}$$

where the neighbourhood u=[ux,uy] is such that $u_{x}$∈[−R,R] and $u_{y}$∈[−R,R].

The time-surface Sk(u,p) associated with the event $e_{k}$ can be obtained by applying either an exponential or linear decay kernel of time-constant τ to the time context $T_{k}$. In this work, the linear decay was used and it is described as shown in Equation (3):

(3) $$S_{k}\left(u,p\right)=\left\{\begin{array}{cc} 1-\frac{T_{i}\left(u,p\right))}{\tau }, & \mathrm{if}\;T_{i}\left(u,p\right)<\tau \\ 0, & \mathrm{otherwise}\; \end{array}\right.$$

Once the time-surface for the incoming event has been created, it is compared with other time-surfaces, known as prototypes, which have been trained previously [28,36]. This set of trained prototypes are stored and used for composing a layer. These prototypes/patterns are learnt using an online clustering method, which can be used for event-driven processing. A layer can have a different number of prototypes N, radius R and time constant **τ**. HOTS proposes the concatenation of several these layers in order to perform a more complex classification among a set of hierarchical features.

**Figure 3:**
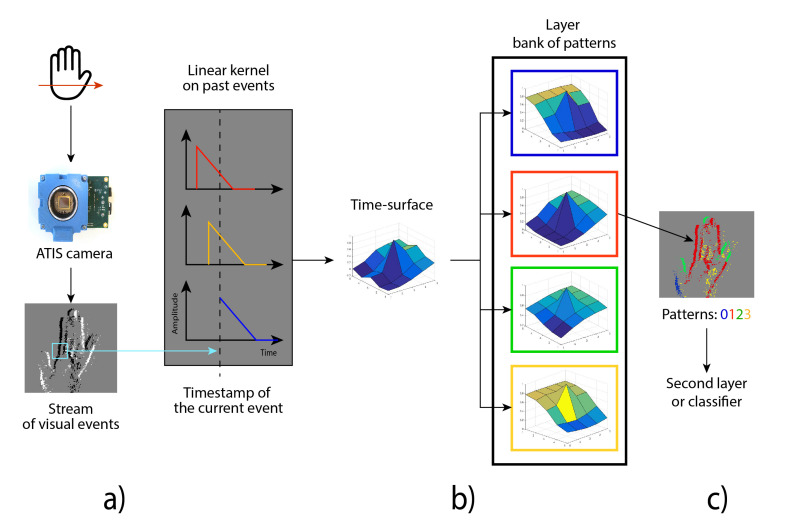
Example of a HOTS layer processing workflow. An input stimulus is processed by the sensor, sending a stream of events with (x,y) addresses and an ON/OFF polarity (**a**). The timestamp context of the incoming event is processed by applying a linear decay, creating the time-surface (**b**). Using the Euclidean distance, the time-surface is compared with the bank of prototypes; the closest one will send out an event with the same (x,y) but with the corresponding ID of the prototype. Finally, the events are sent out to another layer or integrated over time in order to generate a histogram, which is then processed by a classifier (**c**).

For each layer, the time-surface created from an input event is compared with its bank of prototypes, in order to get the feature that best matches the generated time-surface. A cost function, such as the Euclidean distance or cosine distance, can be applied for this searching. The output of each layer is an event with the same (x,y) address and timestamp, but adding the ID of the matching prototype *c* and removing its previous polarity $p_{k}$. Therefore, pk=c, with the event encoding a pattern instead of an ON/OFF polarity. The output event can be used to feed a second layer that processes the event in a similar way, but the second layer combines the features of a previous layer. Otherwise, the output events can be integrated over time, generating a histogram of activated patterns that can be used to feed a classifier. Figure 3 represents the computation process for a one-layer HOTS.

### 2.3. System Architecture

The design presented is a fully digital system that constructs the time-context for an incoming event and implements the HOTS surface generation, the Euclidean distance with the prototype surfaces, the histogram generation and its comparison to perform the classification. The architecture has three different interfaces: two AER interfaces for the four-step asynchronous handshaking protocol, which are used to send and receive signals between neuromorphic systems, and a 32-bit digital interface to configure the system through a host microcontroller. The system includes a fixed-point square-root circuit [37] and can be configured dynamically. The time-context is created by computing the differences between stored timestamps and incoming event’s timestamps. The time-surface is then generated by applying a linear decay to the time-context. Next, the time-surface is compared with stored pattern prototypes, looking for the most similar one. Subsequently, the ID of the closest prototype (pattern) is sent to a histogram generator module, which creates a histogram of activated patterns from event IDs. After a period of time, the generated histogram is compared with trained histograms, in order to obtain the corresponding feature, sending out the classification result through an AER bus. The following subsections describe the functional blocks of the implementation and the processing pipeline in detail.

#### 2.3.1. Time-Surface Generator

The time-surface generator module is in charge of computing the time context and applying the linear decay to the incoming event neighbourhood. The event timestamp has a resolution of 32 bits. In this design, we use a 32-bit counter that assigns the timestamp to each incoming event. The timestamps are stored in an embedded block-RAM (BRAM) memory, whose depth is equal to the sensor resolution. We used 128 × 128 pixels resolution.

**Figure 4:**
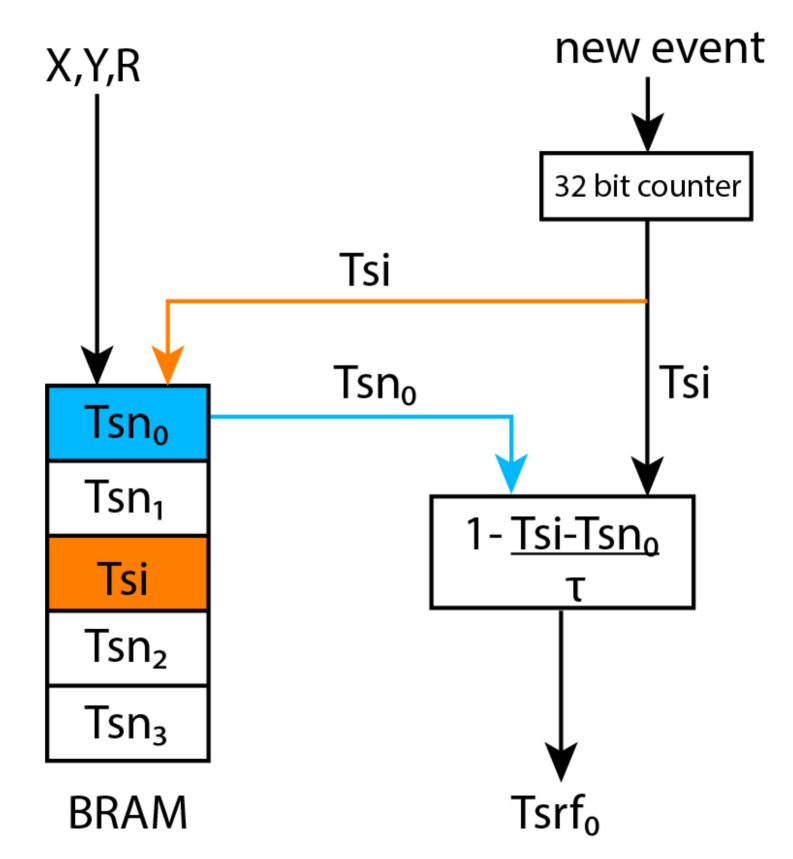
Time-surface generator module workflow.

When an input event arrives the square neighbourhood with dimension (2R+1)×(2R+1) is read event by event, computing the difference between the incoming event timestamp and the stored timestamps (time-context) and applying the linear decay using the τ constant Equation (3). The result is the time-surface value of the pixel addressed by the incoming (x,y) event. Figure 4 represents the workflow of this module, where the timestamp of the incoming event (**Tsi**) is stored and updated in block-RAM, and the neighbour timestamps (**Tsn**) are read sequentially to compute the time-surface of the corresponding pixel (**Tsrf**), following Equation ( 3).

#### 2.3.2. Euclidean Distance Estimator

The Euclidean distance is defined as the distance segment between two points *q* and *p*, as shown in Equation (4). Considering each time-surface and each prototype value as points in space, the Euclidean distance can be computed as the square root of the sum of the square differences between the prototype values and time-surfaces.

**Figure 5:**
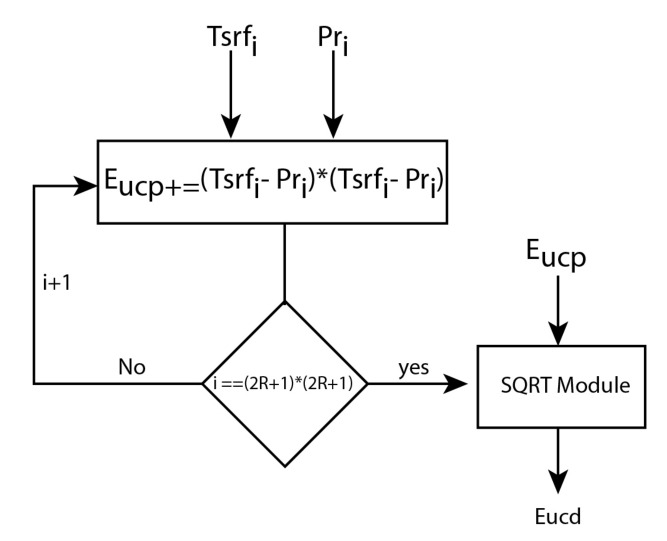
Euclidean distance estimator module workflow.

The Euclidean distance estimator (EDE) module receives the time-surface values of each processing event and computes the Euclidean distance with a stored prototype. When this module receives a time-surface of an event, it reads the prototype value of the spatial position of the incoming time-surface, and then it computes the difference between the two values. The square of the difference is computed and accumulated, as shown in Figure 5.

(4) $$\begin{array}{cc} \; & d\left(p,q\right)=\sqrt{{\left(p_{1}-q_{1}\right)}^{2}+{\left(p_{2}-q_{2}\right)}^{2}+{\left(p_{n}-q_{n}\right)}^{2}}\\ \; & d\left(p,q\right)=\sqrt{\sum_{i=1}^{n}{\left(p_{i}-q_{i}\right)}^{2}}b) \end{array}$$

The square root is computed when the sum of all the differences has been obtained (Equation (4)), i.e., when all the events of a neighbourhood have been processed. The square root module implements the non-restoring square root algorithm [38]. This algorithm computes the square-root of a non-negative number using a sequence of addition/subtraction and bit-shift operations. Due to the simplicity of the performed operations, this algorithm is an efficient way to implement the square-root computation in VLSI systems.

The number of EDE modules depends on the size of the bank of prototypes. EDE modules are individual processing units which work in parallel to process the Euclidean distance of different prototypes at the same time. These modules compute the product of the Euclidean distance, accumulating the result for all the neighbourhood. Then, the square root is computed by the non-restoring square-root module (NR-SQRT), which performs the non-restoring square root algorithm. Time-surface generators and EDE modules work in a pipeline way. Therefore, when a Euclidean module is computing the difference of a neighbour, the time-surface generator module is computing the time-surface for the following neighbour.

Once the computation of the Euclidean distance is performed, the minimum of the resulting Euclidean distances is extracted, and the polarity of the input event is changed with the ID of the corresponding prototype. Then, this event is sent out through the AER interface. The architecture implemented in this work has one time-surface generator, eight EDE modules that match the number of prototypes of the network, with their corresponding NR-SQRT, and the module that obtains the minimum.

#### 2.3.3. Histograms Generator and Comparator Module

**Figure 6:**
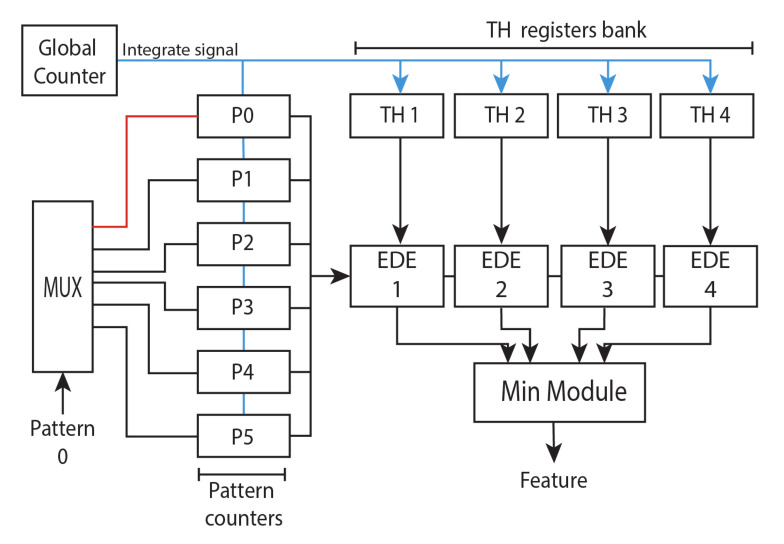
Histograms comparator module (HGCM). The blue signal is asserted when the global counter reaches the integration time, given by the τ value. At that moment, the content of the Pattern counter, which is the histogram of the activated features, is compared with the trained histograms (TH).

The output events encode a pattern within its (x,y) address, instead of an ON/OFF polarity, being integrated for a period of time that matches the τ value of the last layer. In this design, the output events histogram is generated through several counters that increment their value with the arrival of a pattern event. These counters are called Pattern counters. The pattern counters count the number of patterns produced after the comparison with the prototypes, the number of counters is equal to the number of patterns in the network. Each pattern counter represents the activation of a pattern, and they compose the histogram of activated patterns, where each bar’s value is given by the content of the corresponding pattern counter. The histogram of activated patterns is compared with trained histograms (TH). These histograms are stored in a bank of registers. After the integration period is met, a global counter asserts the integrate signal, computing the nearest neighbour algorithm between histograms using the Euclidean distance through EDE modules explained before, and resetting the counters for a new histogram integration while the system is still receiving events. However, the previous EDE takes the data pixel by pixel, since the time-surface value is needed; the EDE implemented in this module processes the square differences for all the columns of the histograms in one clock cycle, reducing the latency, and then the square root is computed. After the Euclidean distance is computed, the classification result corresponds to the closest histograms and it is sent out through AER bus. Figure 6 shows an example of the Histograms generator and comparator module (HGCM) for 5 different patterns and 4 features to be classified.

#### 2.3.4. Hardware Implementation

The design was described as a RTL with System Verilog language and synthesized for a Zynq-7100(xc7z100-2) MMP platform, from AVNET^®^, using Vivado 2016.4 from XILINX ^®^. This platform contains a Programmable System on Chip (PSoC) with: a Dual ARM^®^ Cortex^™^-A9 MPCore, which is called processing system (PS), and a Kintex-7 FPGA, called programmable logic (PL), with 444 K logic cells and an 755 embedded BRAM blocks in the same chip. The FPGA HOTS (F-HOTS) architecture can work with a maximum clock frequency of 100 MHz. Although the design was synthesized for a large platform, due to the available AER interface, the system can fit in a smaller FPGA, such as the one available at the Zynq-7020. Table 1 presents the percentage of the total resources consumed by PS and PL in Zynq-7100 and 7020 FPGAs for a 16-bit computation resolution.

**Figure 7:**
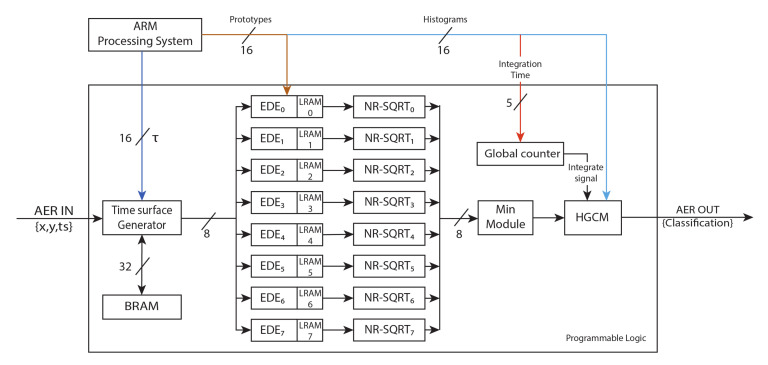
F-HOTS global architecture. The ARM processor configures the parameters and the prototypes of each EDE module. The time-surface generator module creates a time-surface from the incoming event received from the input AER bus (AER IN). Each partial time-surface result is processed by the eight EDE module; then, after processing the histograms, the classification result is sent through the output AER bus (AER OUT).

The whole platform architecture, which is shown in Figure 7, including the PS and the PL requires a power consumption of 1.6 W. For our implementation, the ARM processors need 1.533 W and the remaining 77 mW are consumed by the FPGA logic. These power consumptions were measured with Xilinx power tool after the implementation, assuming a toggle rate of 50% of the signals, which is higher than that of normal operations.

The ARM is in charge of configuring the layer with the different prototype parameters (R, τ ), whereas AER interfaces communicate with neuromorphic sensors by sending or receiving events. In this design, a custom developed board called dock-SoC was used [39], which adapts the I/O pins of Zynq-7100 to AER interface.

## 3. Experimental Set-Up and Results

This section presents the results of a one-layer HOTS architecture. A multilayered implementation could obtain better accuracy results, but it would increase the computation, and thus the needed resources and power consumption, which could be critical for an embedded system. Therefore, the performance for a one layer network deployed with a small number of prototypes is measured. The parameters of the network are: τ=10 ms, R=2 and N=8.

**Figure 8:**
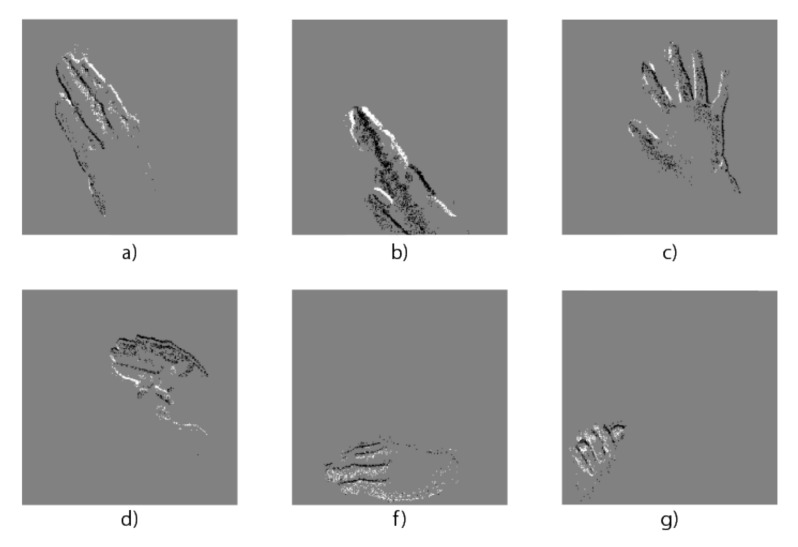
Hand gestures from (**a**–**g**): Left, Right, Hello Hand, Up, Down, Select.

We used a novel dataset called NavGestures-sit. This dataset has 6 hand gestures of 304 × 240 resolution: Right, Left, Up, Down, "Hello-hand" and Select, as shown in Figure 8. It was first used to test the network in [36]. Therefore, in this network an event can encode 8 different patterns and it can classify these 6 gestures. The purpose of our work is to compare the computation accuracy of that software implementation [36] against the design proposed in this article, running the same network in hardware.

**Figure 9:**
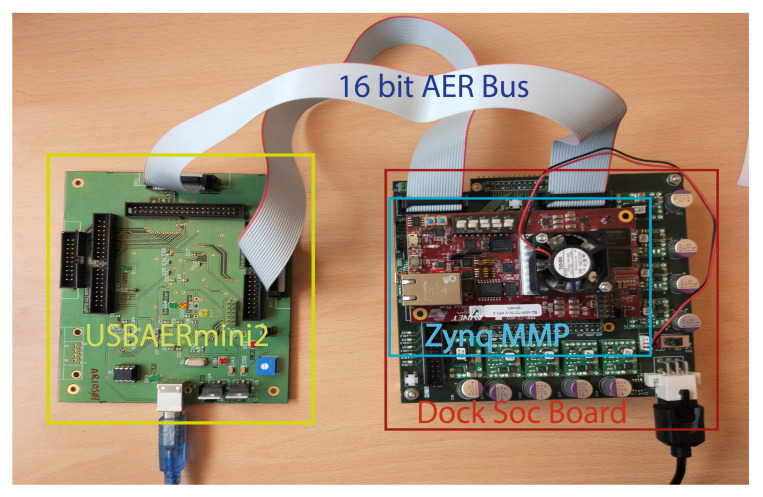
Experimental Setup. **Left**: USBAERmini2 that sends and receives events from FPGA. **Right**: Zynq MMP board with F-HOTS architecture implemented.

The experimental setup is shown in Figure 9. It consists of an AERtool, called USBAERmini2 [40] that sequences events from a computer through USB packets. These events are sent using an AER interface to the Zynq, where the events are processed. Output events are collected by the USBAERmini2 board [40] through its monitor port and they are sent to jAER [35] software. The aim of this experiment, apart from testing the computation accuracy with the NavGestures-sit, is to characterize the system’s behaviour, measuring the latency, the input event maximum throughput and the maximum number of operations computed per second (OP/s). The addresses of events from *NavGestures-sit* were scaled to 128 × 128 resolution to fit in the AER bus. The experiments was divided into two: (1) the accuracy loss test, which computes the error produced in pattern classifications, and (2) a performance test against different input throughput in order to characterize the system.

### 3.1. Loss Test

In fixed-point operations it is normal to lose some precision due to several factors, such as bit truncation or resolution. The accuracy loss obtained by the architecture after processing the dataset using different fixed points resolutions was measured. The computation resolutions used in this experiment were 1632 and 64 bits in **Q_n,__m_** notation, where *n* bits were for the integer part and *m* for the decimal part. In this work, *n* corresponds to the bits of the upper half of the resolution, whereas *m* is the lower-half bits; e.g.,: for 16 bits, *n* is the 8 most significant bits, and *m* is the 8 least significant bits. FPGA circuit classification errors were compared with the software classification result in order to measure the error produced in computation. The average accuracy loss obtained for *NavGestures-sit* for each resolution was 1.2%, 0.78% and 0.4%, respectively, with respect to the classification obtained in software implementation presented in [36]. Table 2 presents the accuracy results obtained for each bit resolution.

Nevertheless, increasing the bit resolution does not significantly affect the accuracy, as is shown in Table 2. However, even if the bit resolution increases, the accuracy loss does not decrease significantly and it would imply more hardware resources.

Table 3 shows the FPGA resources for each platform with different bit resolutions. BRAM resources are not affected by the different resolutions, since their capacity only depends on the sensor spatial resolution and the word width of the timestamps. Both factors remain constant despite the changes. However, LUT increases its consumption, due to the growth in the size of the buses in the design. Thus, combinational blocks also become more complex with wider bit resolution. In spite of this fact, when the prototypes word resolution is higher, since LUTRAM memories need to store them, they consume more resources. Although LUT and LUTRAM have no significant consumption with respect to the total, since bit width has increased, the computation becomes more complex and, therefore, more DSPs are needed, maintaining the same number of clock cycles. Table 3 shows that, for each scenario, hardware resources do not exceed the total in the FPGA. However, for a future multilayer implementation, the number of available DSP blocks could not be enough for smaller devices, such as Zynq-7020, using 32 or 64-bit resolutions.

**Figure 10:**
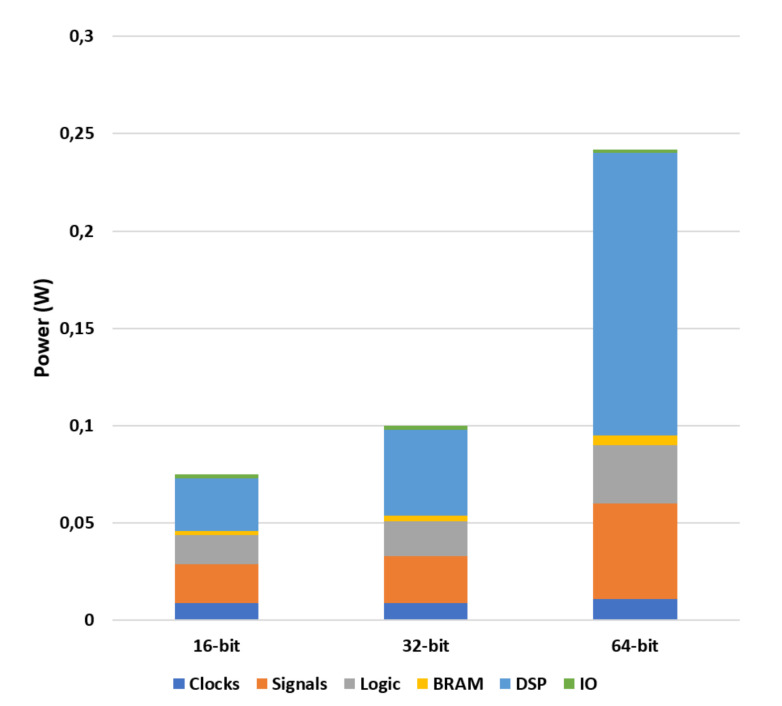
Power consumption of FPGA components for each bit resolution.

The power consumption depends mostly on the word width and DSPs used, thus an increment of bit resolution directly affects both resources. Figure 10 shows the power consumption for each different component of the FPGA divided by resources. The estimated logic power consumption for each different resolution is: 77 mW for 16 bits, 99 mW for 32 bits, and 199 mW for 64 bits.

Therefore, 16-bit resolution is the best option, since the accuracy loss is not significant, and the power consumption is low due to the low use of DSPs. These facts make the 16-bit (Q_n,__m_) resolution implementation ideal for a future multilayer version for small embedded systems.

### 3.2. Performance Test

**Figure 11:**
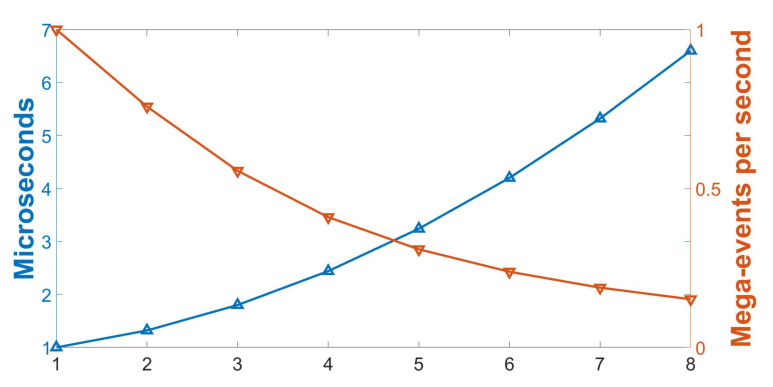
**Left axis**: Processing time per event with different radii. **Right axis**: Evolution of mega-events per second for each different radius.

The latency of the system depends on the square radius of the surfaces ((2R + 1) × (2R + 1)) to be processed. The larger R value, the higher the latency, since more memory transfers are needed. On the other hand, a smaller R implies that the memory bottlenecks are reduced. In this experiment, we measured the processing time for an event for different R values, from 1 to 8. Apart from the processing time, in neuromorphic systems it is important to maintain the supported throughput (Ev/s) from the sensors, since it determines the throughput that the system is able to compute. However, the input stimulus must be faster, to test the behaviour of the system in the worst case. Therefore, the input stimulus selected for this test is different from that in the previous test. A dot turning at 2000 rpm captured with a DVS is now used. Complex computation is centered in time-surface generation and comparison with the bank of prototypes to generate the pattern of incoming events. Therefore, the latency and the input throughput depends on how fast the system is able to generate pattern events. Histogram generation and comparison does not significantly affect the latency and input throughput, since the new events can be processed in pipeline while histograms are being compared to obtain the classification result. In this test, for six objects to classify, the HGCM takes 0.5 μs. Figure 11 shows that R affects both the processing time and the input throughput. Regarding the plot shown in Figure 11, the smallest radius to be processed is for R = 1. This scenario represents the best case, as fewer pixels are processed. The latency obtained for this scenario is 0.5 μs (input throughput of 2 Mev/s). On the other hand, the worst scenario is for the maximum radius of 8 for this architecture, since this performs maximum memory accesses, increasing the latency to 6 μs (input throughput of 0.16 Mev/s).

Another important factor to consider for hardware HOTS implementation is the number of operations per second. The time-surface generator module (TSG) computes a division and a subtraction for each pixel in the square neighbours of radius R, whereas each Euclidean estimator module (ESM) computes two subtractions, one multiplication and one addition for each pixel in the square neighbour and number of prototypes (N) in parallel. Finally, the non-restoring square module (NR-SQRT) computes one addition/subtraction and a shift operation. The total number of operations performed for this architecture is expressed in Equation (5), where **R** is the radius, **N** is the number of prototypes and **T** is the time to process an event:

(5) $$Op/s=\frac{\left(\overset{\overset{\mathrm{Square}\mathrm{neighbourhood}}{︷}}{\left(2R+1)\times (2R+1\right)}\times \left(\overset{\overset{\mathrm{TSG}}{︷}}{2}+\overset{\overset{\mathrm{ESM}}{︷}}{4}\times N\right)\right)+\overset{\overset{\mathrm{NR}-\mathrm{SQRT}}{︷}}{2}\times N}{T}$$

**Figure 12:**
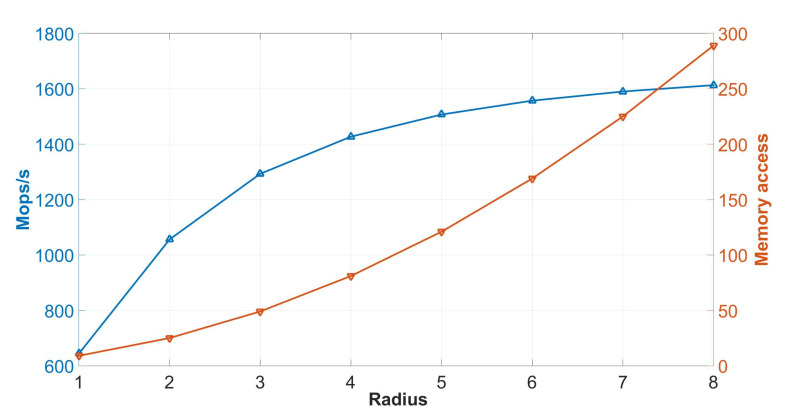
**Left axis**: Mops/s performed with different radii, at a frequency of 100 MHz. **Right axis**: memory accesses performed.

Figure 12 shows the number of mega-operations per second (Mops/s) and the number of memory accesses for different kernel radius. Figure 12 shows how computation increases for different radius sizes below 6. At this point, computation reaches its limit at 1.6 GOps/s. In other words, memory access increases computation as more pixels are processed, thus more operations are performed. However, for a radius of 6, the performance does not depend on memory bandwidth anymore, as it depends on computation resources reaching the computation peak.

## 4. Discussion and Conclusions

FPGAs parallelism is used to increase the speed of complex algorithms, such as Convolutional neural networks [39] or Spiking convolutional neural networks [41]. In addition with its reprogrammable nature, FPGAs are ideal for the implementation of any kind of algorithm in real time, such as HOTS [28]. This work presents a VLSI architecture for FPGA to accelerate the HOTS algorithm. The system was tested with a gesture recognition dataset [42], obtaining an accuracy loss of 1.2% from the algorithm implemented in [36]. The estimation of the power consumption is 77 mW with Xilinx X-Power, working with a frequency of 100 MHz and implying a 50% toggle rate. The system is presented as a new hardware approach for visual pattern recognition in event-based processing, using the novel concept of time-surface, which works directly on inter-event time intervals.

In [43], an event-based gesture recognition application is implemented in the IBM TrueNorth chip using spiking convolutional neural networks (SCNN). This approach also considers the timing information of events, and works with the DVS gesture dataset. It obtains an accuracy of 94.59% and 96.49% for 10 and 11 categories, respectively, with a power consumption of 178.8 mW. Although the accuracy obtained is better than ours, with 4–5 more categories classified, this solution requires a large number of neurons, which implies higher power consumption than our system. In [44], a convolutional neural network for poker cards symbol recognition is implemented. Its maximum accuracy obtained for 4 classes is 96%, and the minimum power consumption is 7.7 mW, but incoming events cannot be processed in real time for that accuracy. This comparative is summarized in Table 4.

The presented system resolves single HOTS layers. A multi-layer HOTS architecture requires more memory resources, since each layer needs its own memory in principle. This is because output events timestamps of the prototype bank must be stored in memory for each prototype, as it is performed in the time-surface generator module. On the other hand, the BRAM memory used in this work has the same dimension as the sensor, in order to store each incoming event. However, neuromorphic vision sensors produce a sparse output, which implies that a large part of the memory is unused. One possible solution to reduce memory consumption is to implement a different memory model that only stores events during a period of time (such as cache memory), reducing memory size and allowing the implementation of a multi-layer version in small FPGA/ASIC platforms. With this memory architecture, our system can be improved by adding more computation modules. Another proposal could be to replace time-surface generator BRAMs with LUTRAMS to decrease the processing time, since LUTRAM memory has a latency of 1 clock cycle instead of 2 clock cycles of BRAM. However, both solutions would have a great impact on logic element resources of the FPGA.

## Figures and Tables

**Table 1:** PS + PL Resource Use for 16-bit resolution.

	Zedboard (xc7020clg482)	Zynq7000 (xc7z100ffg2)
**LUT**	8313/53,200 (15.6%)	8351/277,400 (3%)
**LUTRAM**	2879/17400 (16.5%)	2872/108,200 (2.6%)
**FF**	5627/106,400 (5.2%)	6092/54,800 (1.1%)
**DSP**	46/220 (20%)	46/2020 (2%)
**BRAM**	18/140 (12.8%)	18/755 (2%)

**Table 2:** Accuracy comparison with different numerical precision

	Maro et al. [36]	Q8.8	Q16.16	Q32.32
**NavGestures-sit**	94.5%	93.3%	93.72%	94.1%

**Table 3:** Programmable logic resources as a function of numerical precision.

Zynq7000 (xc7z100ffg2)			
Resolution	Q8.8	Q16.16	Q32.32
LUT	3%	4.22%	4.68%
LUTRAM	0.31%	0.36%	1.67%
FF	0.34%	0.38%	0.48%
DSP	2%	3.23%	6.92%
BRAM	2.12%	2.12%	2.12%
**Zedboard (xc7020clg482)**			
Resolution	Q8.8	Q16.16	Q32.32
LUT	15.6%	16.7%	22.01%
LUTRAM	7.43%	8.41%	10.37%
FF	1.62%	1.88%	2.41%
DSP	20.2%	34.09%	64.45%
BRAM	11.43%	11.43%	11.43%

**Table 4:** Comparison with prior work.

	Platform	Algorithm	Features	Accuracy	Power Consumption (mW)
**This work**	FPGA	HOTS	6	93.3%	77
**Amir, A et al [31]**	IBM-TrueNorth	SCNN	10/11	94.59%/96.49%	178.8
**Camuñas-Mesa et al [44]**	FPGA	SCNN	4	96%	7.7
